# Decreased aggressive care at the end of life among advanced cancer patients in the Republic of Korea: a nationwide study from 2012 to 2018

**DOI:** 10.1186/s12904-024-01459-7

**Published:** 2024-06-25

**Authors:** Sara Kwon, Kyuwoong Kim, Bohyun Park, So-Jung Park, Hyun Jung Jho, Jin Young Choi

**Affiliations:** 1 Department of Hospice & Palliative Service, Hospital, National Cancer Center, Goyang, Republic of Korea; 2 National Hospice Center, National Cancer Control Institute, National Cancer Center, 323 Ilsan-Ro, Ilsandong-Gu, Goyang, Gyeonggi-Do Republic of Korea; 3 Graduate School of Cancer Science and Policy, National Cancer Center, Goyang, Republic of Korea; 4 Division of Cancer Control and Policy, National Cancer Control Institute, National Cancer Center, Goyang, Republic of Korea; 5 Division of Cancer Prevention, National Cancer Control Institute, National Cancer Center, Goyang, Republic of Korea

**Keywords:** Aggressiveness, End-of-Life care, Advanced cancer, Hospice

## Abstract

**Background:**

This study aimed to investigate the trends of aggressive care at the end-of-life (EoL) for patients with advanced cancer in Korea and to identify factors affecting such care analyzing nationwide data between 2012 to 2018.

**Methods:**

This was a population-based, retrospective nationwide study. We used administrative data from the National Health Insurance Service and the Korea Central Cancer Registry to analyze 125,350 patients aged 20 years and above who died within one year of a stage IV cancer diagnosis between 2012 and 2018.

**Results:**

The overall aggressiveness of EoL care decreased between 2012 and 2018. In patients’ last month of life, chemotherapy use (37.1% to 32.3%; *p* < 0.05), cardiopulmonary resuscitation (13.2% to 10.4%; *p* < 0.05), and intensive care unit admission (15.2% to 11.1%; *p* < 0.05) decreased during the study period, although no significant trend was noted in the number of emergency room visits. A steep increase was seen in inpatient hospice use in the last month of life (8.6% to 26.6%; *p* < 0.05), while downward trends were observed for hospice admission within three days prior to death (13.9% to 11%; *p* < 0.05). Patients were more likely to receive aggressive EoL care if they were younger, women, had treatment in tertiary hospitals, or had hematologic malignancies. In the subgroup analysis, the overall trend of aggressive EoL care decreased for all five major cancer types.

**Conclusion:**

The aggressiveness of EoL care in stage IV cancer patients showed an overall decrease during 2012–2018 in Korea.

**Supplementary Information:**

The online version contains supplementary material available at 10.1186/s12904-024-01459-7.

## Introduction

The last few decades have seen a worldwide growing interest in quality of life among cancer patients, along with the expansion of palliative care. Although certain new treatments have succeeded in prolonging the survival of advanced cancer patients [1, 2], the majority of these patients still face end-of-life (EoL) issues as a consequence of their disease progression. Therefore, improving the quality of life of these patients at the end of their life is an important issue. This quality of life essentially includes reducing physical, psychological, social, and spiritual suffering [3], along with sensitive and timely communication. In this regard, integrating palliative care in EoL care has proved to enhance the quality of life for advanced cancer patients by reducing the use of futile medical treatments near death, such as chemotherapy, cardiopulmonary resuscitation (CPR), or admission to an intensive care unit (ICU) [4–6]. Therefore, palliative care provision is an important factor in enhancing EoL among advanced cancer patients; however, even with the spread of palliative care [7–9], the practice of using aggressive care near death showed inconsistent trends among different countries.

Recently, there were two significant institutional changes in the Republic of Korea, which are associated with EoL care. First, the Ministry of Health and Welfare (MoHW) has promoted hospice palliative care (HPC) as a national policy since the 2000s. After several years of a pilot project, MoHW officially designated a hospice palliative care unit, which is eligible for government subsidies. In 2015, the National Health Insurance (NHI) initiated a reimbursement plan for designated HPC units. The provision and utilization of HPC gradually increased as awareness of the EoL issue improved among the public [10, 11]. Second, as social interest escalated in life-sustaining treatment and self-determination of patients, the Act on Hospice and Palliative Care and Decisions on Life-sustaining Treatment for Patients at the End of Life was established in 2016, and came into effect in 2018 [12]. The purpose of this act is “To protect the dignity and value of human beings by assuring the best interests of the patients and by respecting their self-determination” [12].

While several studies have examined the changes in practices of EoL care, especially the use of aggressive care in Korea [5, 13–16], most of the study populations were limited to patients from a single institution or with a single type of cancer. This study aimed to analyze the recent trend of aggressive care at the EoL among patients with different types of advanced cancer using nationwide data and the demographic and clinical factors associated with aggressive care near death.

## Methods

### Data sources and study population

In this retrospective, population-based study, we used a matched database from two data sources: the National Health Information Database held by the National Health Insurance Service (NHIS), and the Korea Central Cancer Registry (KCCR). We identified stage IV cancer diagnoses from the KCCR data and used the NHIS data to identify the medical use patterns and level of aggressive care provided to patients diagnosed with stage IV cancer.

**Fig. 1:**
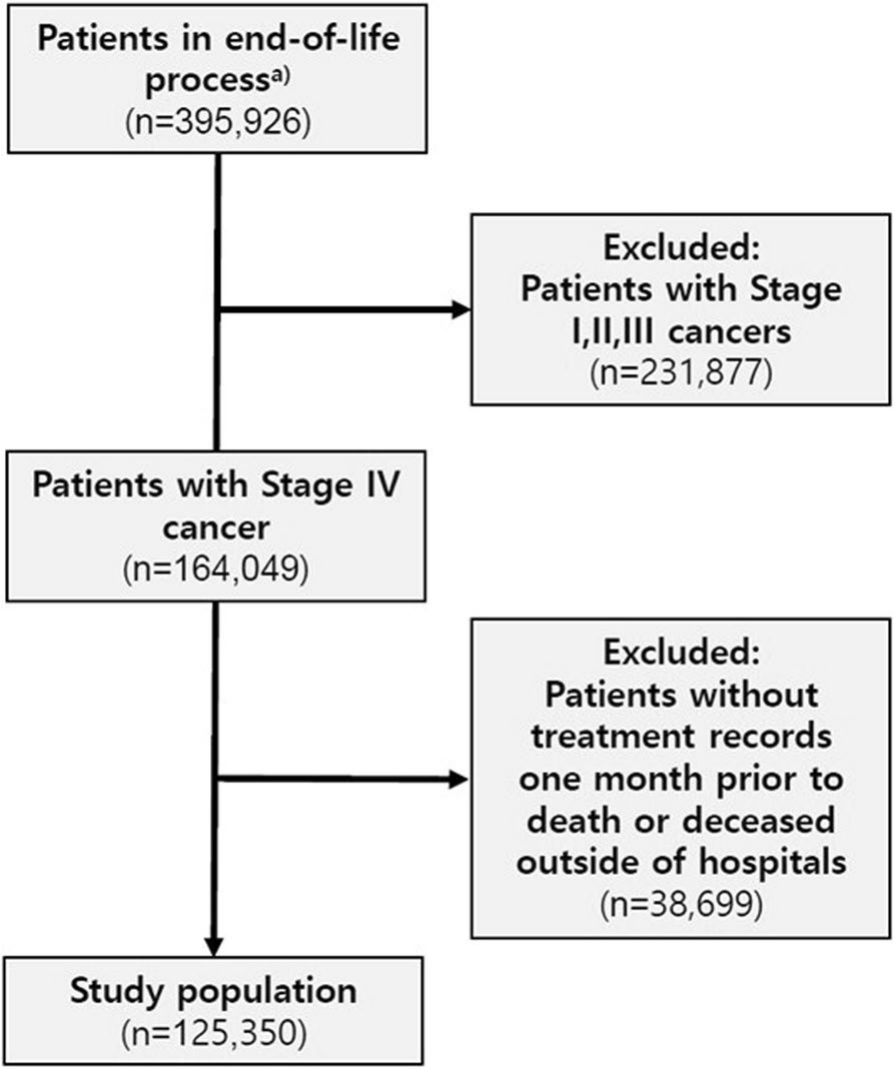
Patient selection process. **a** Patients who died within 1 year of first cancer diagnosis between 2012 and 2017 identified in the NHIS-KCCR matching database. NHIS, National Health Insurance Service; KCCR, Korea Central Cancer Registry

The inclusion criteria were patients over 20 years of age who died within one year of a stage IV cancer diagnosis. Such patients were selected by identifying those who had a main diagnosis of cancer on an NHIS claim within one month of death. Patients who did not have NHIS claim records within one month of death and those who died outside the hospital were excluded. Finally, 125,350 of 395,926 individuals were analyzed as satisfying this condition (Fig. 1).

### Main outcome measure

EoL was defined as one month before death, similar to previous studies [17, 18]. The indicators of aggressive EoL care were adopted from Earle et al. [19] and other studies on futile life-sustaining treatment for terminal cancer patients [20].

The main indicators were obtained from the billing codes in the NHIS and defined as follows: (1) chemotherapy in the last month of life, (2) CPR in the last month of life, (3) ICU admission in the last month of life, (4) emergency room (ER) visits more than twice in the last month of life, (5) utilization of inpatient hospice in the last month of life, and (6) hospice admission within three days prior to death.

Information regarding the use of chemotherapy, CPR, ICU admission, and ER visits in the last month of life was extracted from both outpatient and inpatient claims data. Based on the inpatient claims, utilization of inpatient hospice care in the last month of life, and instances of hospice admission of less than three days were identified. The claims data for hospice and palliative care insurance have been available since 2015.

We studied the demographic and clinical factors that affect these aggressiveness indicators at the EoL. We also investigated the trends of these indicators for all types of cancer, and the five non-sex-specific cancers with the highest crude mortality rates in Korea [21] (lung, liver, colorectal, pancreatic, and gastric).

### Explanatory variables

Patients were grouped into five categories according to age (under 50, 50–59, 60–69, 70–79, and over 80 years), and the cancer type was classified according to the main types of cancer death in Korea: lung, colorectal, gastric, pancreatic, liver, biliary, hematologic, or other cancers according to the International Classification of Disease 10th revision (ICD-10) codes. Income quartiles were categorized based on income level using health insurance premium levels in the 20-ranked quantile measure. Places of death were classified into tertiary referral hospitals, general hospitals, and local clinics, according to their size and function. The regions of the place of death were classified according to the administrative division of Korea.

### Statistical analysis

Descriptive analyses were used to investigate the demographic and clinical characteristics of the patients. Joinpoint regression was used to analyze and identify significant trends in aggressive care over time for all cancers and individual cancers. Multivariable logistic regression analyses were performed to estimate the association between patient characteristics and indicators of aggressive EoL care or hospice utilization. We adjusted for determinants including age, sex, cancer type, income, institution of death, and geographic region of death. All statistical analyses were performed using SAS software version 9.4 for Windows (SAS Institute Inc., Cary, NC, USA), and a P value less than 0.05 was considered statistically significant.

## Results

### Patient characteristics

According to the screening procedure shown in Fig. 1, a total of 125,350 patients met the eligibility criteria. Table 1 shows their demographic and clinical characteristics. The mean age of the patients was 67.6 years old, with 79,235 (63.2%) of them being male. Lung cancer was the most common cancer, followed by colorectal, gastric, pancreatic, and liver cancer. As for the income quartile, the lowest group was 24,593 (19.6%) and the highest group was 47,354 (37.8%). The most common places of death were tertiary and general hospitals (72.3%). In addition, as shown in Table S1 (Online resource 1), there were no significant differences in these characteristics by year.

**Table 1:** Characteristics of Patients (*N* = 125,350)

	*n*	%
**Total**	125,350	100
**Average Age ± SD**	67.6 ± 13.3	
**Age, years**		
50	11,493	9.2
50–59	20,888	16.7
60–69	30,126	24.0
70–79	39,809	31.8
≥ 80	23,034	18.4
**Sex**		
Male	79,235	63.2
Female	46,115	36.8
**Cancer type**		
Lung	38,302	30.6
Colorectal	13,636	10.9
Gastric	13,571	10.8
Hematologic	12,930	10.3
Pancreatic	11,969	9.6
Liver	10,482	8.4
Biliary	5510	4.4
Other	18,950	15.1
**Income quartile**^**a**^		
1 (lowest)	24,593	19.6
2	23,697	18.9
3	29,706	23.7
4	47,354	37.8
**Place of death, institution type**		
Tertiary referral hospital	42,748	34.1
General hospital	47,905	38.2
Local clinic	34,697	27.7
**Year of Death**		
2012	8554	6.8
2013	16,154	12.9
2014	19,360	15.4
2015	20,762	16.6
2016	22,410	17.9
2017	23,531	18.8
2018	14,579	11.6

*SD* Standard Deviation

^a^ Income quartile by the health insurance premium level in 20-ranked quantile measure

### Indicators of aggressive EoL care

**Fig. 2:**
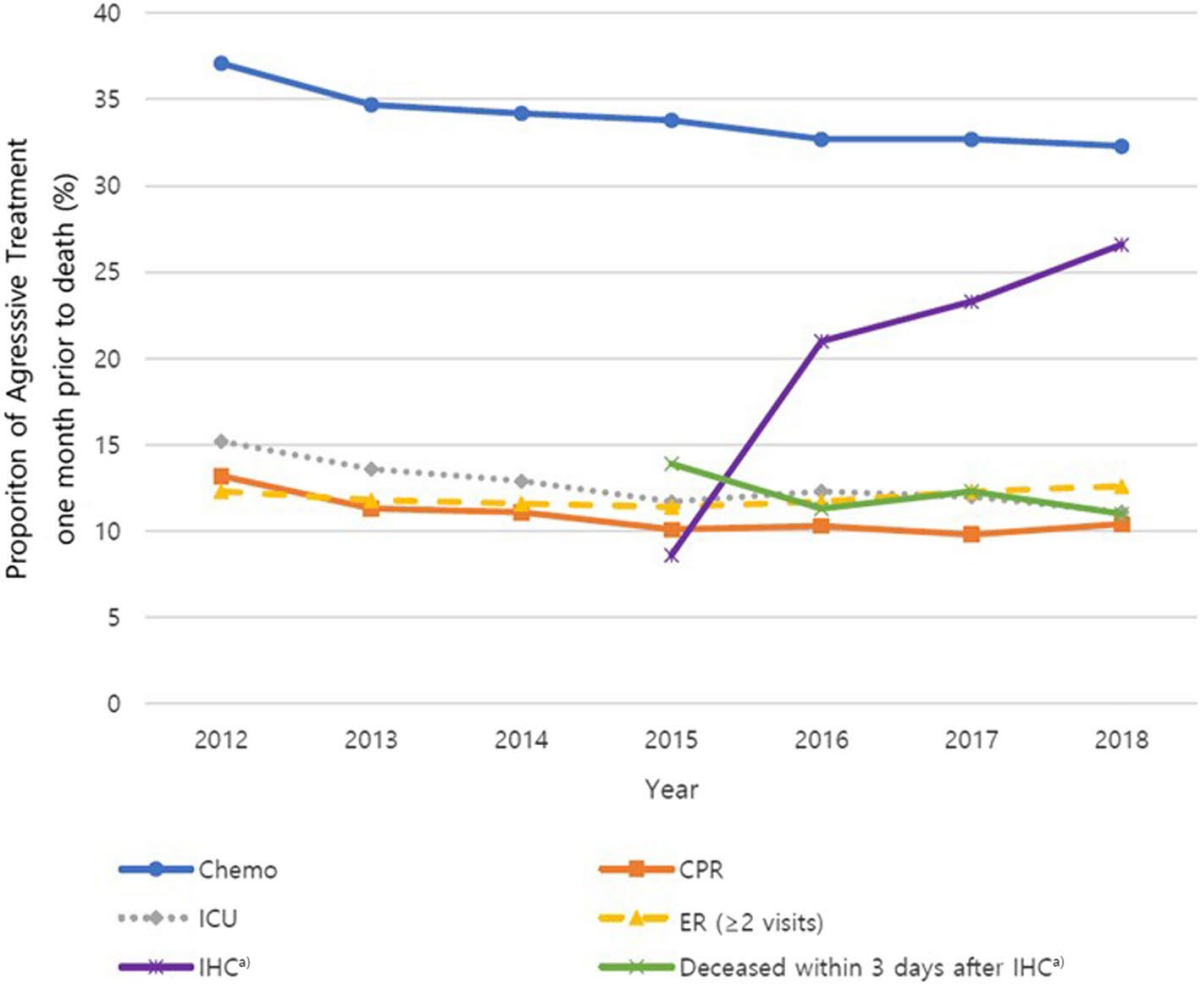
Trends in indicators of aggressive care for all cancers during 7-year study period. Chemo, chemotherapy; CPR, cardio-pulmonary resuscitation; ICU, intensive care unit; ER, emergency room; IHC, inpatient hospice care. ^a)^ The claim data for hospice and palliative care use insurance had been available since 2015

Figure 2 depicts trends in the aggressiveness of EoL care between 2012 and 2018. Chemotherapy use (37.1% to 32.3%; *p* < 0.05), CPR (13.2% to 10.4%; *p* < 0.05) and ICU admission (15.2% to 11.1%; *p* < 0.05) in the last month of life decreased over the study period. ER visits more than twice in the last month of life slightly decreased between 2012 and 2015 (12.3% to 11.4%), but increased thereafter (11.4% to 12.6%).

Since 2015, utilization of inpatient hospice care in the last month of life increased steeply until 2018 (8.6% to 26.6%; *p* < 0.05), and downward trends were observed for hospice admission within three days before death (13.9% to 11%; *p* < 0.05). The trend analysis with the annual percent change (APC) is shown in Table S2. Chemotherapy, CPR, and ICU admission in the last month of life decreased significantly over the study period (*p* < 0.05).

### Predictors of aggressive EoL care

Table 2 shows a multivariable logistic regression analysis predicting aggressive care and hospice utilization in the last month of life. We found that as age increased, experiencing chemotherapy (OR, 0.99 for each year; 95% CI, 0.99–1.00), receiving CPR (OR, 0.99; 95% CI, 0.99–1.00), ICU admission (OR, 0.99; 95% CI, 0.99–1.00), ER visits more than twice (OR, 0.98; 95% CI 0.98–0.99), inpatient hospice use (OR, 0.99; 95% CI, 0.99–1.00) in the last month of life, and hospice admission within three days before death (OR, 0.99; 95% CI, 0.99–1.00) decreased significantly. Women received more chemotherapy (OR, 1.19; 95% CI 1.16–1.22) and CPR (OR, 1.23; 95% CI 1.18–1.28) than men in their last month of life. Women were also more likely than men to be admitted to the ICU in their last month of life (OR, 1.18; 95% CI 1.13–1.22), and more likely to visit the ER more than twice in the last month of life (OR, 1.10; 95% CI, 1.06–1.15). However, women’s inpatient hospice use in the last month of life (OR, 0.85; 95% CI 0.82–0.88) was lower than that of men, and hospice admission within three days before death (OR, 1.16; 95% CI, 1.05–1.28) was higher. With respect to cancer type, hematologic malignancies had a higher risk than lung cancer in all indicators except ER visits and inpatient hospice use. Among the places of death, chemotherapy, CPR, and ICU admission in the last month of life were performed more frequently in tertiary hospitals than in general hospitals and local clinics, and inpatient hospice use was lower. There were no significant associations between income level and aggressiveness of EoL care.

**Table 2:** Multivariable logistic regression analysis predicting aggressive care and utilization of hospice (*N* = 125,350)

	Chemotherapy in the last month of life		CPR in the last month of life		ICU admission in the last month of life		≥ 2 ER visits in the last month of life		Utilization of inpatient hospice		Hospice admission ≤ 3 days before death	
Factor	OR^a^	95% CI	OR	95% CI	OR	95% CI	OR	95% CI	OR	95% CI	OR	95% CI
**Age, years**	0.99*	0.99–1.00	0.99*	0.99–1.00	0.99*	0.99––1.00	0.98*	0.98–0.99	0.99*	0.99–1.00	0.99*	0.99–1.00
**Sex**												
Male	ref		ref		ref		ref		ref		ref	
Female	1.19*	1.16–1.22	1.23*	1.18–1.28	1.18*	1.13–1.22	1.10*	1.06–1.15	0.85*	0.82–0.88	1.16*	1.05–1.28
**Cancer type**												
Lung	ref		ref		ref		ref		ref		ref	
Colorectal	0.58*	0.56–0.61	0.67*	0.62–0.72	0.82*	0.76–0.87	0.85*	0.80–0.91	1.62*	1.53–1.71	0.85*	0.72–0.99
Gastric	0.64*	0.61–0.67	0.61*	0.56–0.65	0.66*	0.62–0.71	0.92*	0.87–0.98	1.28*	1.20–1.35	0.91	0.77–1.08
Pancreatic	0.91*	0.87–0.95	0.46*	0.42–0.50	0.53*	0.49–0.58	1.06	1.00–1.13	1.46*	1.38–1.55	0.94	0.79–1.11
Liver	0.76*	0.73–0.80	0.47*	0.43–0.51	0.72*	0.67–0.77	1.19*	1.12–1.27	1.01	0.94–1.08	1.05	0.86–1.27
Biliary	0.77*	0.72–0.82	0.49*	0.44–0.56	0.59*	0.53–0.65	1.06	0.97–1.15	1.29*	1.19–1.40	0.69*	0.53–0.91
Hematologic	1.05*	1.00–1.09	2.51*	2.38–2.65	2.73*	2.59–2.87	0.77*	0.72–0.82	0.40*	0.36–0.44	1.57*	1.24–2.00
Other	0.68*	0.65–0.70	0.95	0.90–1.01	1.07*	1.01–1.13	0.89*	0.84–0.94	1.42*	1.35–1.50	0.98	0.84–1.13
**Income level**^b^	0.99	0.98–1.00	0.99	0.97–1.01	1.00	0.98–1.01	1.03*	1.02–1.05	1.04*	1.02–1.05	1.02	0.98–1.07
**Place of death, institution type**												
Tertiary referral hospital	ref		ref		ref		ref		ref		ref	
General hospital	0.67*	0.65–0.69	0.66*	0.64–0.69	0.90*	0.86–0.93	1.07*	1.03–1.11	1.65*	1.58–1.73	0.95	0.84–1.09
Local clinic	0.33*	0.32–0.34	0.20*	0.18–0.21	0.17*	0.16–0.18	0.48*	0.45–0.50	1.54*	1.46–1.61	1.19*	1.04–1.37

*CPR* cardiopulmonary resuscitation, *ICU* intensive care unit, *ER* emergency room, *OR* odds ratio, *CI* confidence interval, *ref*. reference

^a^Adjusted odds ratios derived from multivariable logistic regression model

^b^Income quartile by the health insurance premium level in 20-ranked quantile measure

^*^P < 0.05

### Aggressive EoL care by type of cancer

**Fig. 3:**
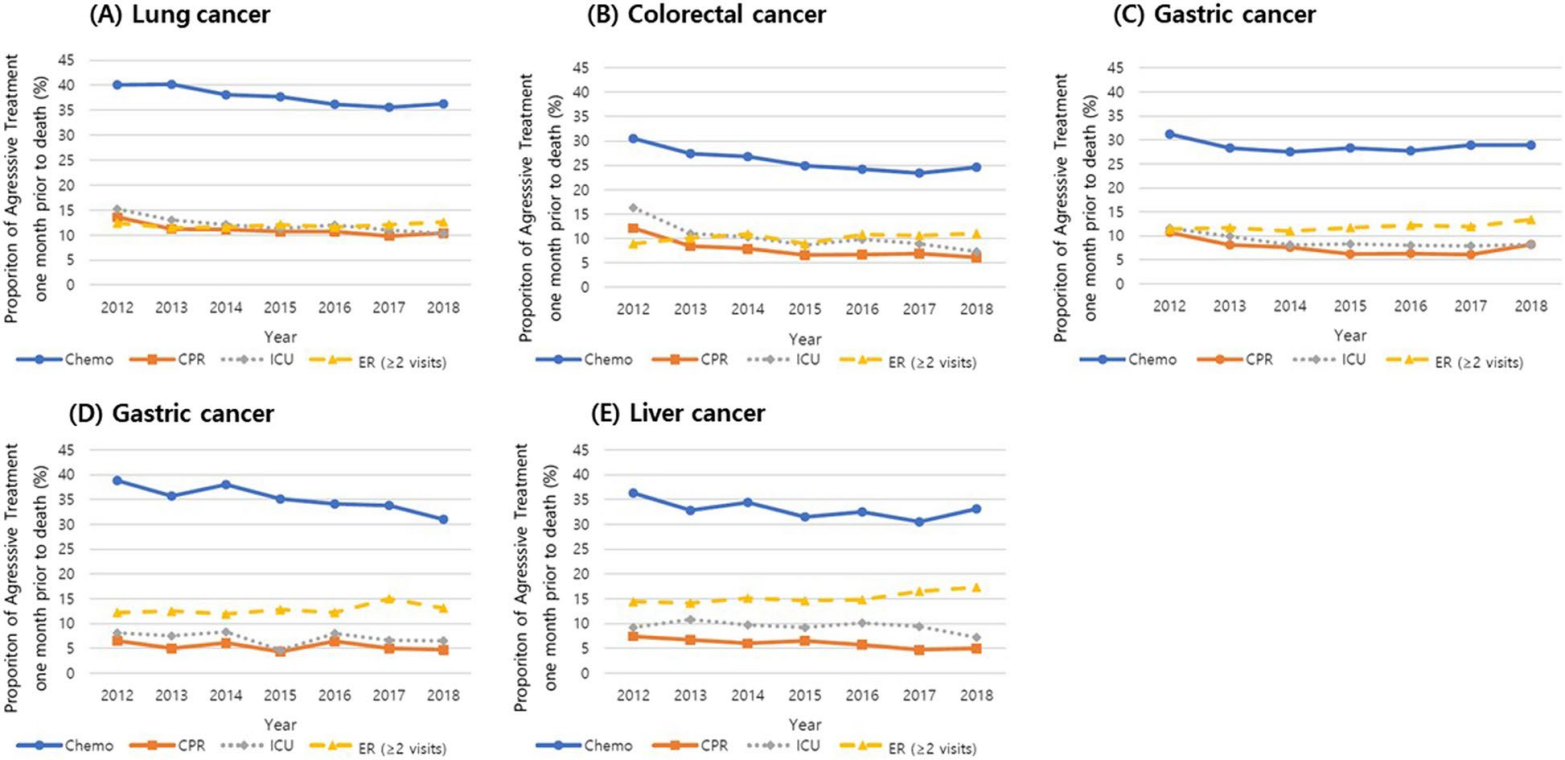
Trends in indicators of aggressive care for each type of cancer during 7-year study period. **A** lung, **B** colorectal, **C** gastric, **D** pancreatic, **E** liver cancer. Chemo, chemotherapy; CPR, cardio-pulmonary resuscitation; ICU, intensive care unit; ER, emergency room

Aggressive care at the EoL tended to decrease during the study period for all the five major cancer types (Fig. 3). The proportion of patients receiving chemotherapy in the last month of life decreased during the study period, from 40.1% to 36.3% in lung cancer, 30.5% to 24.6% in colorectal cancer, 31.2% to 28.9% in gastric cancer, 38.8% to 31% in pancreatic cancer, and 36.3% to 33.1% in liver cancer. The proportion of patients receiving CPR and being admitted to an ICU in the last month of life also decreased for all five major cancers.

## Discussion

This is the first study to analyze the trends of EoL care aggressiveness in patients with stage IV cancer using national data. We found an overall decrease in aggressive treatment at the end of life for stage IV cancer patients in Korea between 2012 and 2018, including the rates of receiving chemotherapy, ICU admission, and CPR. During the study period, there was a trend of increase in use of inpatient hospice and decrease of late hospice admission. This trend was consistent among five cancers of highest mortality in Korea.

Over the study period, the aggressiveness of EoL care in patients with stage IV cancer decreased. This may be related to the increase in the number of hospice institutions and beds in Korea and the increase in public awareness of hospice and EoL care between 2012 and 2018. According to a recent report by the MoHW and the National Hospice Center (NHC), the number of hospice institutions in Korea increased from 56 in 2012 to 158 in 2018, and the number of hospice beds increased steadily from 893 to 1,542. In addition, the hospice utilization rate of cancer patients also showed a steady increase from 11.9% in 2012 to 22.9% in 2018, which is consistent with the increase in inpatient hospice use found in this study [10]. According to another report by the MoHW and NHC, public awareness of hospice or palliative care increased from 71.6% in 2012 to 92.4% in 2018 [22, 23]. Additionally, social discussions on EoL care increased during the study period, resulting in the establishment of the Act on Hospice and Palliative Care and Decisions on Life-sustaining Treatment for Patients at the End of Life in 2016, which was subsequently implemented in 2018. This institutional change could have influenced the trend of aggressive care for terminally ill patients [24, 25].

Over the years during the study period, the rates of receiving chemotherapy, ICU admission, and CPR decreased, inpatient hospice use increased, and late hospice admission decreased. Similar patterns of aggressive EoL care were observed in studies with Korean pediatric patients [16] and Qatari patients who died between 2009 and 2013 [26]. These results are contrary to studies showing an increase in aggressive care provided in the United States [17], Canada [18], and Taiwan [27] between the mid-1990s and early 2000s.

Among the indicators, the proportion of ER visits more than twice in the last month of life did not show any significant change. Differences in predisposition may occur because ER visits are not always caused by cancer itself or by cancer-related complications but are influenced by comorbid conditions. As such, we found it to be less useful as an indicator of aggressive EoL care, similar to observations by Earle et al. [28].

In previous studies on the trend of aggressive care at the end of life, the related causes of increased aggressiveness in care included low hospice access and patients’ and families’ attitudes toward hospice care and chemotherapy [17]. In contrast, the Qatar study found that the opening of the palliative care unit and implementation of a do not attempt resuscitation (DNAR) policy were related to a decrease in aggressive care [26]. Similarly, a Korean study with children [16] described a decrease in aggressive care due to changes in attitude toward EoL. Another study also suggested that the Hospice-Palliative Care Act may be associated with an increased use of hospice services and a decrease in CPR in EoL [27]. Other studies [13, 16] suggested that changes in patients’ financial burden caused by changes in chemotherapy-related medical insurance policies led to changes in the aggressiveness of EoL care. In Korea, as health insurance was applied to hospice-palliative care in 2015, the financial burden of hospice use decreased, and the number of hospice institutions increased. This development in Korea’s hospice infrastructure and the change in attitude toward hospice and EoL care are thought to be related to the decrease in aggressive care found in this study.

We also found that patient factors such as age, sex, and institution of death, as well as disease characteristics such as cancer type were significant independent predictors of aggressive EoL care. Younger age was a significant independent predictor of aggressive EoL care, consistent with other studies [17, 18, 28]. This is thought to be because the younger the age, the higher the physical tolerability of the treatment, and the higher the expectations of patients, families, and physicians for a full recovery.

Our study also found female patients to receive more aggressive treatment such as chemotherapy, CPR, ER visits, and ICU admissions, while having a lower rate of hospice use, which was inconsistent with most other studies [17, 18, 28]. One study, however, reported higher rates of late-stage chemotherapy in female patients than in male patients [29].

Depending on the cancer type, hematologic malignancies are strongly associated with highly aggressive EoL care and low and late use of hospice care [28, 30]. This was also observed in our study, which is thought to be due to the characteristics of hematologic malignancy, such as a high frequency of hematologic complications and therapeutic optimism based on a plethora of treatment options, which is different from solid cancer [31].

Our findings indicated that receiving care at a tertiary referral hospital at the EoL results in more aggressive care than general hospitals or local clinics. This is consistent with what has been reported in other studies [17, 28] probably because tertiary hospitals are in an environment where active treatment is possible compared with other hospitals.

We performed a subgroup analysis to determine the status and trend of aggressive treatment according to the cancer type. All the five major cancer types showed a tendency toward decreasing EoL care aggressiveness. The proportion of patients receiving chemotherapy showed a steady decrease in all five major cancer types; however, in 2018, the rate of lung cancer patients receiving chemotherapy was higher than that of other cancers, which is related to the emergence of many new treatment options, such as targeted therapy and immunotherapy for lung cancer [32, 33]. However, for colorectal and gastric cancers, the rate of chemotherapy at the end of life was relatively low. This seems to be related to the limited treatment options because traditional cytotoxic chemotherapy is the mainstay of colorectal or gastric cancer treatment despite the development of targeted therapies [14, 34].

There are a few limitations in this study. First, several significant factors related to the use of aggressive care at EoL such as patient’s performance status, comorbidities, awareness of disease status, patient preference for EoL care, and communication on EoL were not included in the analysis. Second, patients without treatment records or who died outside the hospital (*n* = 38,699) were excluded from the analysis. Finally, the differences in illness trajectory between hematologic malignancies and solid tumors need to be considered in analysis.

In conclusion, the EoL care aggressiveness of patients with stage IV cancer showed an overall tendency to decrease during the study period from 2012 to 2018 in Korea, among all five major cancer types. Further studies are needed to identify other factors related to EoL care aggressiveness, and to find out ways to effectively manage these factors to improve quality of life for advanced cancer patients.

### Supplementary Information

Supplementary Material 1.

## Data Availability

The data supporting the findings of this study are obtainable from the NHIS (National Health Insurance Service) and KCCR (Korea Central Cancer Registry). However, access to these data may be restricted due to their usage under approval policy for the current study, making them not publicly accessible. Nevertheless, interested parties can request access to the raw data from the NHIS and KCCR, subject to the approval by the relevant review board upon reasonable inquiries.

## Abbreviations

CPR: Cardiopulmonary Resuscitation
EoL: End-of-Life
ER: Emergency Room
HPC: Hospice Palliative Care
ICU: Intensive Care Unit
ICD-10: International Classification of Disease 10th revision
KCCR: Korea Central Cancer Registry
NHI: National Health Insurance
OR: Odds Ratio
