# Supplementary material for: Decreased aggressive care at the end of life among advanced cancer patients in the Republic of Korea: a nationwide study from 2012 to 2018
Source: BMC Palliat Care. 2024 Jun 25;23:160. doi: 10.1186/s12904-024-01459-7 (PMC11201316; doi:10.1186/s12904-024-01459-7)
Supplement: Supplementary file 1 — Supplementary Material 1. [file 12904_2024_1459_MOESM1_ESM.docx]

**S1 Table.** Characteristics of Patients by Year of Death (N=125350)

|  | 2012 | 2013 | 2014 | 2015 | 2016 | | 2017 | | 2018 | |
| --- | --- | --- | --- | --- | --- | --- | --- | --- | --- | --- |
|  | (n = 8554) | (n = 16154) | (n = 19360) | (n = 20762) | | (n = 22410) | | (n = 23531) | | (n = 14579) |
| **Mean age, years (SD)** | 68.69 (12.91) | 67.34 (13.22) | 67.33 (13.35) | 67.34 (13.34) | | 67.71 (13.38) | | 68.33 (13.15) | | 66.45 (13.23) |
| **Sex (%)** |  |  |  |  | |  | |  | |  |
| Male | 5683 (66.4) | 10495 (65.0) | 12494 (64.5) | 13010 (62.7) | | 14008 (62.5) | | 14741 (62.6) | | 8804 (60.4) |
| Female | 2871 (33.6) | 5659 (35.0) | 6866 (35.5) | 7752 (37.3) | | 8402 (37.5) | | 8790 (37.4) | | 5775 (39.6) |
| **Cancer type (%)** |  |  |  |  | |  | |  | |  |
| Lung | 2588 (30.3) | 5104 (31.6) | 6047 (31.2) | 6460 (31.1) | | 6955 (31.0) | | 7023 (29.8) | | 4125 (28.3) |
| Colorectal | 655 (7.7) | 1613 (10.0) | 1975 (10.2) | 2235 (10.8) | | 2492 (11.1) | | 2630 (11.2) | | 2036 (14.0) |
| Gastric | 980 (11.5) | 1969 (12.2) | 2244 (11.6) | 2250 (10.8) | | 2352 (10.5) | | 2354 (10.0) | | 1422 (9.8) |
| Pancreatic | 1019 (11.9) | 1618 (10.0) | 1848 (9.5) | 2066 (10.0) | | 2106 (9.4) | | 2237 (9.5) | | 1075 (7.4) |
| Liver | 1116 (13.0) | 1621 (10.0) | 1787 (9.2) | 1681 (8.1) | | 1729 (7.7) | | 1769 (7.5) | | 779 (5.3) |
| Biliary | 462 (5.4) | 752 (4.7) | 861 (4.4) | 920 (4.4) | | 958 ( 4.3) | | 1066 (4.5) | | 491 (3.4) |
| Hematologic | 844 (9.4) | 1502 (9.3) | 1862 (9.6) | 2128 (10.2) | | 2373 (10.6) | | 2521 (10.7) | | 1700 (11.7) |
| Other | 890 (10.4) | 1975 (12.2) | 2736 (14.1) | 3022 (14.6) | | 3445 (15.4) | | 3931 (16.7) | | 2951 (20.2) |
| **Income quartile^a^ (%)** |  |  |  |  | |  | |  | |  |
| 1 (lowest) | 1683 (19.7) | 3225 (20.0) | 3838 (19.8) | 4059 (19.6) | | 4352 (19.4) | | 4639 (19.7) | | 2797 (19.2) |
| 2 | 1660 (19.4) | 2986 (18.5) | 3593 (18.6) | 3922 (18.9) | | 4235 (18.9) | | 4466 (19.0) | | 2835 (19.4) |
| 3 | 1964 (23.0) | 3833 (23.7) | 4585 (23.7) | 4834 (23.3) | | 5369 (24.0) | | 5526 (23.5) | | 3595 (24.7) |
| 4 | 3247 (38.0) | 6110 (37.8) | 7344 (37.9) | 7947 (38.3) | | 8454 (37.7) | | 8900 (37.8) | | 5352 (36.7) |
| **Place of death, institution type (%)** |  |  |  |  | |  | |  | |  |
| Tertiary referral hospital | 2794 (32.7) | 5448 (33.7) | 6225 (32.2) | 7341 (35.4) | | 7842 (35.0) | | 7944 (33.8) | | 5154 (35.4) |
| General hospital | 3473 (40.6) | 6374 (39.5) | 7738 (40.0) | 7755 (37.4) | | 8526 (38.0) | | 8806 (37.4) | | 5233 (35.9) |
| Local clinic | 2287 (26.7) | 4332 (26.8) | 5397 (27.9) | 5666 (27.3) | | 6042 (27.0) | | 6781 (28.8) | | 4192 (28.8) |
| **Place of death, region (%)** |  |  |  |  | |  | |  | |  |
| A | 2084 (24.4) | 3915 (24.2) | 4636 (23.9) | 5073 (24.4) | | 5571 (24.9) | | 5668 (24.1) | | 3669 (25.2) |
| B | 1934 (22.6) | 3750 (23.2) | 4562 (23.6) | 4898 (23.6) | | 5336 (23.8) | | 5744 (24.4) | | 3662 (25.1) |
| C | 1448 (16.9) | 2642 (16.4) | 3170 (16.4) | 3416 (16.5) | | 3753 (16.7) | | 3912 (16.6) | | 2485 (17.0) |
| D | 941 (11.0) | 1834 (11.4) | 2117 (10.9) | 2235 (10.8) | | 2322 (10.4) | | 2512 (10.7) | | 1475 (10.1) |
| E | 640 (7.5) | 1184 (7.3) | 1359 (7.0) | 1341 (6.5) | | 1457 (6.5) | | 1486 (6.3) | | 878 (6.0) |
| F | 594 (6.9) | 1121 (6.9) | 1366 (7.1) | 1510 (7.3) | | 1515 (6.8) | | 1629 (6.9) | | 957 (6.6) |
| G | 319 (3.7) | 491 (3.0) | 665 (3.4) | 700 (3.4) | | 762 (3.4) | | 751 (3.2) | | 384 (2.6) |
| H | 210 (2.5) | 459 (2.8) | 525 (2.7) | 592 (2.9) | | 639 (2.9) | | 667 (2.8) | | 395 (2.7) |
| I | 313 (3.7) | 602 (3.7) | 766 (4.0) | 771 (3.7) | | 822 (3.7) | | 881 (3.7) | | 515 (3.5) |
| J | 71 (0.8) | 156 (1.0) | 194 (1.0) | 226 (1.1) | | 233 (1.0) | | 281 (1.2) | | 159 (1.1) |

^a^ Income quartile by the health insurance premium level in 20-ranked quantile measure

SD, standard deviation

**S2 Table.** Trends in Indicators of Aggressiveness during 2012-2018.

|  | 2012 | 2013 | 2014 | 2015 | 2016 | 2017 | 2018 | APC |
| --- | --- | --- | --- | --- | --- | --- | --- | --- |
| Proportion with chemotherapy in the last month of life | 37.1 | 34.7 | 34.2 | 33.8 | 32.7 | 32.7 | 32.3 | -1.7 (-2.9 to -0.5)*** |
| Proportion with CPR in the last month of life | 13.2 | 11.3 | 11.1 | 10.1 | 10.3 | 9.8 | 10.4 | -3.8 (-6.6 to -0.8)*** |
| Proportion with ICU admission in the last month of life | 15.2 | 13.6 | 12.9 | 11.7 | 12.3 | 12 | 11.1 | -4.3 (-6.5 to -2.1)*** |
| Proportion with ≥2 ER visits in the last month of life | 12.3 | 11.8 | 11.6 | 11.4 | 11.7 | 12.3 | 12.6 | 0.6 (-0.4 to 1.6)^a^ |
| Proportion with utilization of inpatient hospice | 0 | 0 | 0 | 8.6 | 21 | 23.3 | 26.6 | 41.8 (-20.1 to 151.7) |
| Proportion with hospice admission ≤3 days before death | 0 | 0 | 0 | 13.9 | 11.3 | 12.3 | 11 | -6.0 (-20.1 to 10.6) |

**P*<0.05, ***P*<0.01, ****P* <0.001.

^a^ Average Annual Percent Change.

APC, annual percentage change; CPR, cardiopulmonary resuscitation; ICU, intensive care unit; ER, emergency room
